# Miscellaneous Intelligence

**Published:** 1820-09-01

**Authors:** 


### Miscellaneous Intelligence.

1. *Tracheotomy.* This operation has lately been twice performed by Mr. Richard Carmichael, of Dublin. In one case, for croup in an adult female, the operation was completely successful. A portion of one of the rings of the trachea was removed, in the manner recommended by Mr. Lawrence. In the other case, the impediment to respiration was occasioned by an abscess which had formed between the œsophagus and cervical vertebræ, and obstructed both passages. If the cause had been ascertained before death, Mr. Carmichael thinks the patient's life might have been saved. The details of these cases, and useful practical remarks on the diseases which formed the subjects of the operations, will be given in the forth-coming volume of the Transactions of the Royal College of Physicians of Ireland.

2. *Seeds of the Colchicum Autumnale.* Dr. W. H. WILLIAMS, of Ipswich, who is already well known to the Profession, as a most intelligent and respectable physician, has published, in the last month's MEDICAL REPOSITORY, an interesting paper on the Effects of the Seeds of the Colchicum Autumnale in Chronic Rheumatism. The preparation he employs is as follows:

℞. Sem. colchici autumnal. siccat. ℥ij.
Vini Hispanici [Sherry] - - Oj.

Digere per dies octo vel decem, subinde agitando, cola, et in vasi probè clauso usui serva.

In adults, he commences with a fluid drachm, in a little aromatic water, once or twice a-day, gradually increasing the dose to three drachms; beyond which he has not had occasion to go. It should be taken two or three hours after breakfast, and repeated at bed-time. Its only sensible operation is relieving the sufferings of the patient, and gently operating on the bowels. Dr. Williams has cured a number of patients labouring under obstinate chronic rheumatism, and entertains sanguine hopes "that we have at length found the happy desideratum, a powerful, yet mild, medicine, capable of substituting calmness, tranquillity, and balmy sleep, in the place of pain, weariness, and restless nights—a renovation of long lost limbs, and comparatively robust health, in lieu of feebleness and emaciation." *—May the anticipations of this worthy physician be realized! The colchicum autumnale abounds in several parts of Suffolk, and grows plentifully in the North and West of England. The seeds should be gathered in the end of June or early in July, carefully dried, and kept in a dry place. Any application to Mr. Fitch, chemist, in Ipswich, will readily obtain the quantity desired. While the medicine is used, it is desirable to avoid flatulent food, especially fish, broths, gruel, milk, puddings, and undressed vegetables; together with a diminution in the usual quantity of beer, tea, coffee, and chocolate. We think the profession indebted much to Dr. Williams for the introduction of a medicine that promises to be so useful in the practice of the healing art.

* London. Med. Repository, August 1820, p. 94.

3. *Extract of Pyrola Umbellata.* We ought, in our last Number, to have acknowledged the receipt of a paper from Dr. Crane, Physician to the Boston Dispensary, containing some interesting cases, where the extract of pyrola umbellata was exhibited in much larger doses than have been recommended, but with very doubtful effects. A few days ago we received, from Dr. Crane, another well-detailed case; but which is too long to insert at present. The result of Dr. Crane's experience, however, may be gathered from the concluding passage in his last letter.

"From what can be drawn from this case, my former conjectures appear to be in a great measure confirmed; viz. That the medicine in question may be given in much larger doses than were first prescribed—that, uncombined with other diuretics, it does not appear to increase the secretion of urine—that, in large doses, it may, in some particular cases, be regarded as an auxiliary to other medicines well known to possess diuretic qualities—and lastly, that in no case ought any reliance to be placed upon its supposed virtues in dropsical affections."

W. CRANE, M. D.

---

4. *Note from* Dr. GEORGE PEARSON *to* Dr. JAMES JOHNSON.

George Street, Hanover Square.

Aug. 7, 1820.

Dear Sir,

I think the Medical Public are greatly in debt to you for your admirable exposition, and judicious pathology of apoplexy, contained in the leading article of the last number of the Medico-Chirurgical Review.

Notwithstanding the minute researches published by Dr. Abercrombie and yourself, unless I have overlooked the record, the important pathological fact—*the ossified condition of the cerebral arteries, especially of the carotids*, has been omitted. I believe the late Mr. Cruickshanks, or Dr. John Hunter, first made public this condition of the arteries in the brain, and I have very often observed it in persons who had died of apoplexies. I have not time to enter into any discussions; but I conceive, that those carried on so *temperately*, yet zealously, between Dr. Abercombie and yourself, must widely spread the most useful practical information. As a mark of my attention, I trouble you with this small contribution.

I remain, dear Sir, with much esteem, your faithful servant,

GEORGE PEARSON.

In returning thanks to Dr. Pearson for his obliging remarks, and Communication, the Editor begs to say, that although ossification of the arteries was not distinctly stated in the Etiology of Apoplexy, yet, among the *exciting* causes of the disease were placed—" those chronic disorganizations which take place within the cranium, and which, by *by obstructing the circulation, compressing the cerebral*

*mass, or otherwise injuring the vital powers of the brain,* excite that state called apoplexy." P. 6.

In an eclectic article, in a Journal of this kind, it is the object rather to delineate the leading principles of pathology and practice than enumerate the particulars; and, on this account, the writer of the article alluded to abstained from detailing the great number of organic lesions of the brain which have been recorded by authors, as predisposing to, or exciting apoplexy.

